# Trastuzumab deruxtecan effectively controlled recurrent ovarian large-cell neuroendocrine carcinoma with low-level HER-2 expression: a case report

**DOI:** 10.3389/fonc.2024.1339806

**Published:** 2024-01-26

**Authors:** Yurou Xing, Yidan Cao, Xin Wu, Yue Chen, Yongsheng Wang

**Affiliations:** ^1^ Thoracic Oncology Ward, Cancer Center, West China Hospital, Sichuan University, Chengdu, Sichuan, China; ^2^ Department of Pathology, West China Second Hospital of Sichuan University/Key Laboratory of Birth Defects and Related Diseases of Women and Children, Sichuan University, Chengdu, Sichuan, China; ^3^ Head and Neck Oncology Ward, Cancer Center, West China Hospital, Sichuan University, Chengdu, Sichuan, China; ^4^ Department of Medical Oncology, Cancer Center, and State Key Laboratory of Biotherapy, West China Hospital, Sichuan University, Chengdu, China; ^5^ Clinical Trial Center, National Medical Products Administration Key Laboratory for Clinical Research and Evaluation of Innovative Drugs, West China Hospital, Sichuan University, Chengdu, China; ^6^ State Key Laboratory Biotherapy, Cancer Center, West China Hospital, Sichuan University, Chengdu, Sichuan, China

**Keywords:** neuroendocrine cancer, ovary, HER2, trastuzumab, ADCS

## Abstract

Large-cell neuroendocrine carcinoma (LCNEC) of the ovary is an extremely rare tumor with invasive clinical behavior and poor outcome. However, there is no consensus on the optimal treatment strategy. Surgery followed by chemotherapy is considered the most common therapeutic option. Here, we report a case of a 55-year-old woman with ovarian LCNEC who relapsed after radical surgery and multiple lines of therapy. The tumor lesions continued to grow, and further immunohistochemistry showed low human epidermal growth factor receptor 2 (HER2) expression. After treatment with the anti-HER2 drug trastuzumab deruxtecan (T-DXd, formerly DS-8201a), the tumor burden was significantly reduced, and the patient achieved a progression-free survival (PFS) of 4 months. Our case provides a potential treatment option for recurrent ovarian LCNEC with low-level HER2 expression.

## Introduction

Ovarian carcinoma is one of the most lethal gynecologic malignancies. Primary ovarian LCNEC is an exceedingly rare malignant tumor of the ovary, accounting for less than 2% of ovarian malignant tumors, with a very poor prognosis (1). It often occurs in middle-aged and elderly women, with abdominal distension and abdominal pain, and lacks other specific symptoms (2, 3). Histopathological examination is an important way to diagnose ovarian neuroendocrine carcinoma. Due to the low incidence of this tumor, there is no standard treatment. Surgery combined with adjuvant chemotherapy is the cornerstone of treatment. HER2 expression has not been reported in ovarian LCNEC. We report the first case of ovarian LCNEC with low HER2 expression after multiline therapy, and significant tumor shrinkage was observed after T-DXd treatment.

## Case report

**Figure 1:**
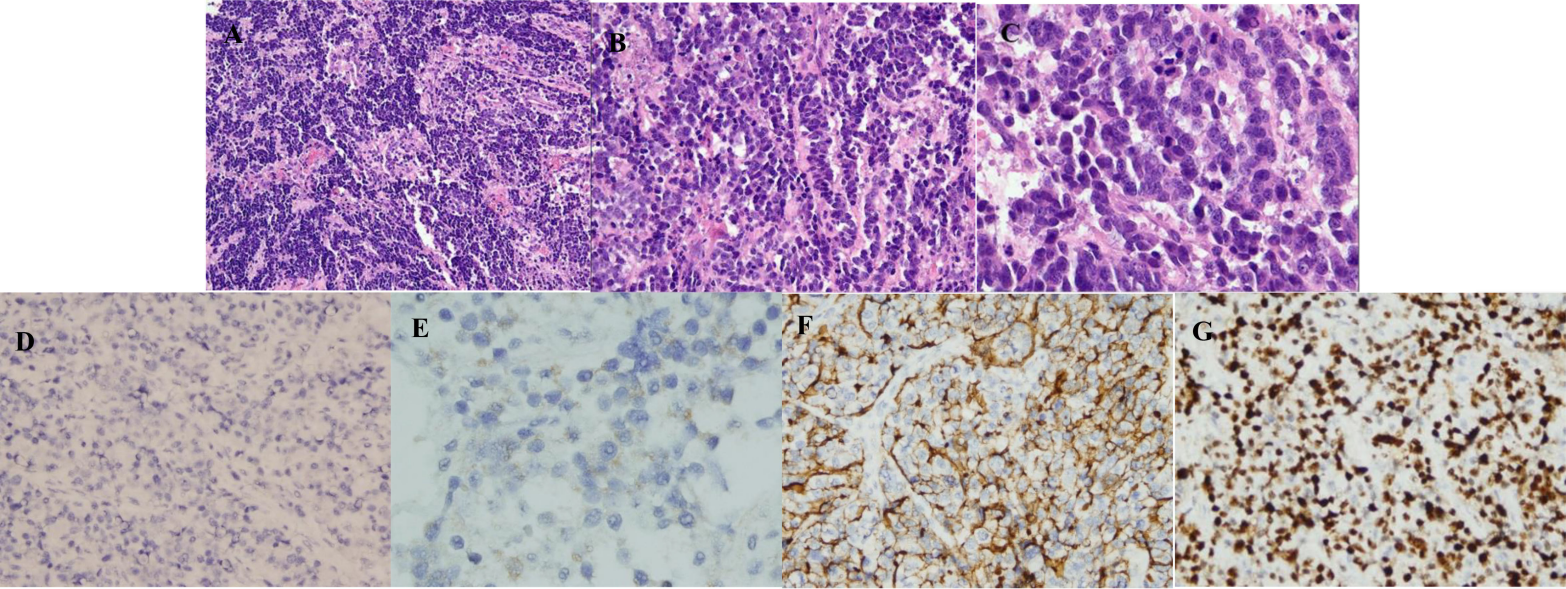
Microscopic pictures. **(A)** Neuroendocrine carcinoma (HE, ×100). Microscopically, the tumor cells were arranged in a diffuse-like, cable-like manner. **(B)** Neuroendocrine carcinoma: (HE, ×200). It is composed of tumor cells with the same size, large nucleus, obvious nucleolus, abundant cytoplasm, and easy to see the mitotic image. **(C)** Neuroendocrine carcinoma (HE, ×400). It is easy to see the mitotic image. **(D)** Chromogranin A is negative in a neuroendocrine carcinoma (×200). **(E)** Syn is focally expressed in a neuroendocrine carcinoma (×400). **(F)** CD56 is expressed in a neuroendocrine carcinoma (×200). (G) Ki-67 is expressed in a neuroendocrine carcinoma, about 85% (×200).

**Figure 2:**
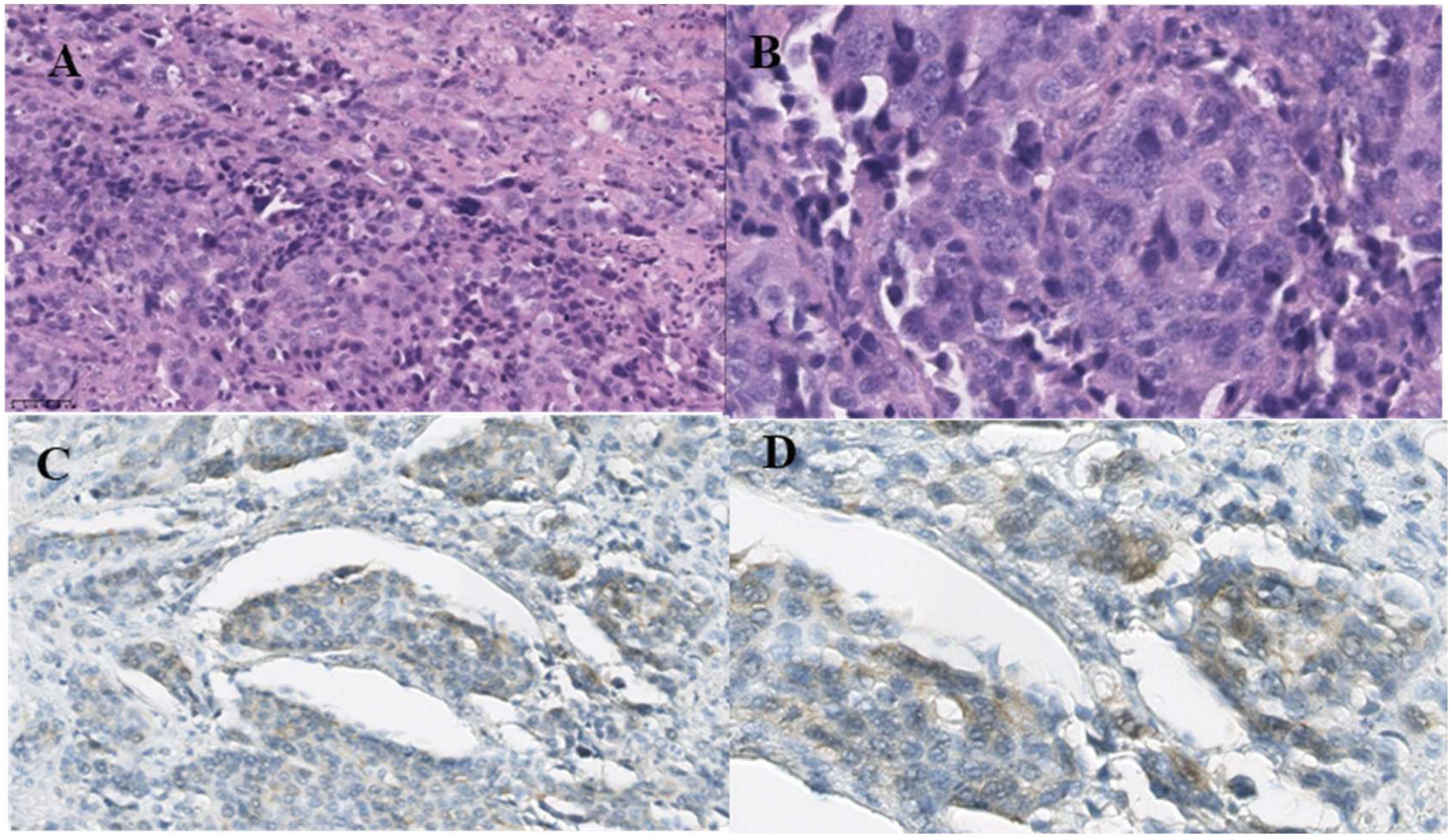
Microscopic pictures. **(A)** Low-power view of poorly differentiated carcinoma of the ovary (HE, ×200). **(B)** High-power view of poorly differentiated carcinoma of the ovary (HE, ×200). **(C)** HER-2 is positive in the carcinoma (×200). **(D)** HER-2 is positive in the carcinoma (×400). The cell membrane of cancer cells is weakly stained.

**Figure 3:**
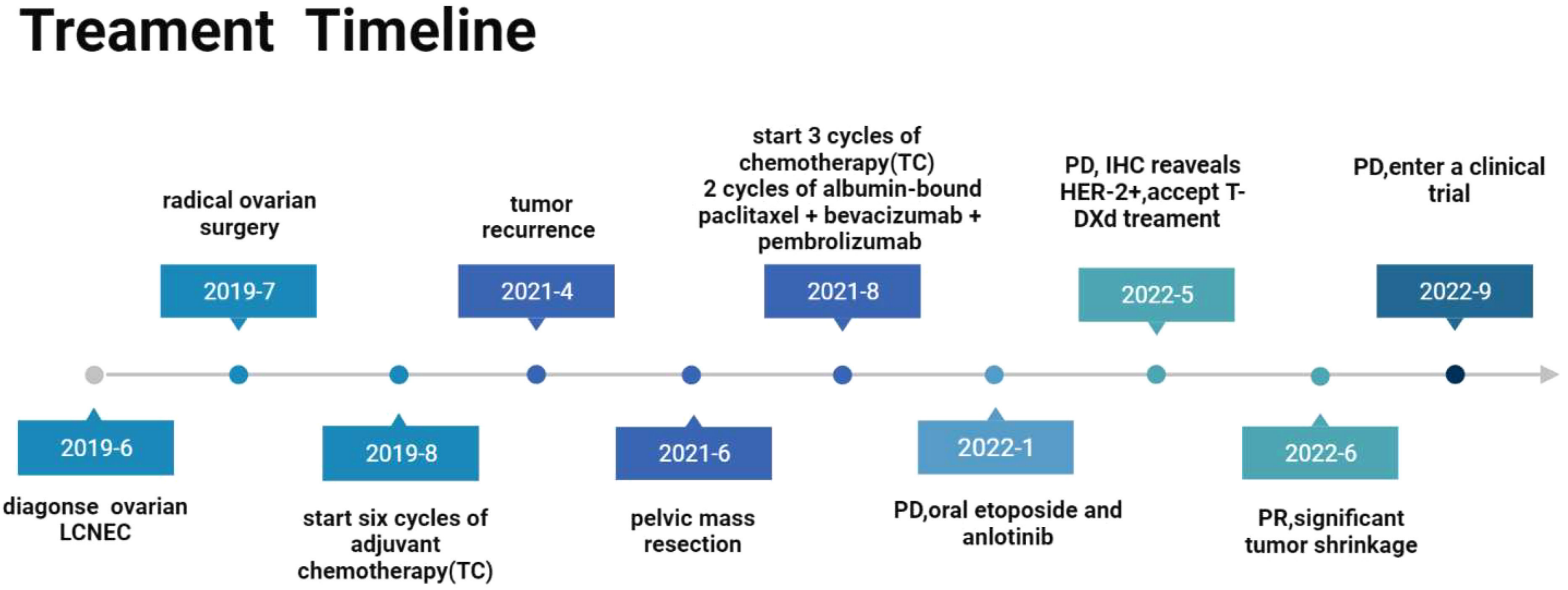
Diagram of the patient’s treatment course. PD, Progressive disease; PR, Partial response.

**Figure 4:**
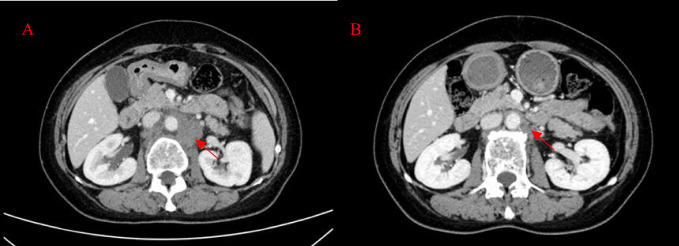
Patient’s CT imaging before and during T-DXd treatment. **(A)** The para-aortic lymph nodes were enlarged (2022-5-13); **(B)** The para-aortic lymph nodes decreased after T-DXd treatment (2022-6-29).

A 55-year-old woman sought consultation for lower abdominal pain and distension with no noted triggers in June 2019. She had no remarkable past medical history or family history. B-ultrasonography and computed tomography (CT) indicated ovarian space occupation, and the tumor marker CA125 was found to be greater than 400 U/ml. The patient underwent ovarian radical surgical treatment on July 3, 2019. Histopathological and immunohistochemistry findings were suggestive of ovarian LCNEC (Stage IIIA) with positive expression for synaptophysin, CD56, and ki-67 (**Figure 1**). She received six cycles of adjuvant albumin-bound paclitaxel plus carboplatin chemotherapy and was regularly reviewed after surgery. Unfortunately, a repeat CT scan performed in April 2021 revealed a pelvic mass, which was suggestive of tumor recurrence. Pelvic mass resection was subsequently performed in June 2021. The immune index test results were positive (PDL1 TPS>1%). After debulking surgery, the patient was treated with 3 sessions of albumin-paclitaxel combined with carboplatin chemotherapy and then switched to paclitaxel combined with pembrolizumab and bevacizumab due to platinum drug allergy. Immunotherapy was stopped because of the development of immune-related hepatitis. CT reexamination after 5 cycles of treatment showed disease progression. The patient started on etoposide and anlotinib tablets in January 2022. However, the growth of the tumor was not controlled. Then, the patient intended to undergo radiotherapy, but radiotherapy was stopped after the first dose of radiotherapy due to high side effects. This patient underwent multiple lines of therapy, and the disease continued to progress. Therefore, we performed further pathological examination, which indicated that the tumor was poorly differentiated carcinoma, and immunohistochemical results showed CgA(-), Syn(-), CD56(-), and her2(1+) (**Figure 2**). Based on this result, the patient accepted T-DXd beginning in May 2022 (**Figure 3**). A review after 2 cycles of treatment indicated that metastatic lymph nodes were significantly reduced, and blood examination of CA125 gradually decreased. She had significant pain relief during the time, and the curative effect evaluation at this time showed a partial response. Nevertheless, CT examination on 22 September 2022 revealed that the pelvic lesions were enlarged, and the disease progressed again (**Figure 4**). The progression-free survival was 4 months with T-DXd. After this progression, she was screened for enrollment in a clinical trial in September 2022.

## Discussion

Ovarian neuroendocrine tumors can be divided into low-grade neuroendocrine tumors and high-grade neuroendocrine carcinomas. LCNEC is a high-grade neuroendocrine carcinoma with aggressive behavior and poor prognosis (4, 5). Neuroendocrine carcinomas generally arise from neuroendocrine cells of the neuroendocrine system. The origin of ovarian large cell neuroendocrine cells is unclear. Three theories have been proposed: the first is derived from neuroendocrine cells in the normal epithelium of the female reproductive tract, the second is transformed from genetically activated nonneuroendocrine cells, and the third is from stem cells with differentiation potential (6).

Epithelial ovarian tumors tend to occur in older women, while LCNEC occurs in a wide range of ages (18-80 years old) (7, 8). Clinically, most patients present with nonspecific symptoms such as abdominal pain, abdominal distension, vaginal bleeding, and pelvic mass. Ovary LCNEC usually presents as a pelvic mass, and CT and MRI often have no specific manifestations. Thus, it is difficult to diagnose LCNEC by imaging alone (6). Due to the aggressiveness of this disease, recurrence or metastasis can occur in a short time. The most common metastatic site of ovarian LCNEC is the abdominal and pelvic cavity, and metastasis to other organs is relatively rare (9). Microscopically, the tumor cells were large, large nucleus, oval in appearance, with areas of necrosis and increased mitotic figures (10). Positive neuroendocrine markers such as chromogranin, CD56, synaptophysin and neuron-specific enolase are often used as the basis for diagnosis (11). In addition, several cases have reported that ovarian LCNEC is usually mixed with other epithelial tumors (3, 8). The differentiation of LCNEC from other ovary tumors is mainly based on pathological examination, which are confirmed by positive immunohistochemical results of neuroendocrine markers. LCNEC can be distinguished from primary and secondary ovarian neuroendocrine tumors. Primary ovarian carcinoid is often characterized by low mitotic activity. Small cell neuroendocrine carcinoma of the ovary is small in size. Metastatic neuroendocrine carcinoma requires finding the primary focus. Additionally, CA125 is increased in epithelial ovarian cancer and correlates with treatment response and tumor progression (12). However, in ovarian LCNEC, the change in CA125 is not completely correlated with the disease condition (7). Retrospective studies are necessary to clarify the reliable prognostic factors of ovarian LCNEC.

There are limited data on treatment options for ovarian LCNEC. Bilateral salpingo-oophorectomy and total hysterectomy are the most common surgical procedures in the literature (13). Chemotherapy is often required after tumor resection. The choice of chemotherapy regimen can refer to epithelial carcinoma of the ovary or pulmonary neuroendocrine carcinoma, including paclitaxel-carboplatin, cisplatin-cyclophosphamide and etoposide-cisplatin (7, 14). Radiotherapy is also the choice for some advanced neuroendocrine tumors (15). However, those treatment options are limited. Patients with LCNEC of the ovary have a poorer survival rate than those with other ovarian carcinomas. The overall 5-year survival rate is 34.9%, and the median OS for ovarian LCNEC is 20 months (5, 9). HER2 amplification accounts for about 3.8% of all gynecological tumors, with uterine serous carcinoma, and mucinous ovarian cancers being the most common (16). HER2 expression has never been reported previously in ovarian LCNEC.

In our case, the tumor was unresponsive to multiple therapies and progressed rapidly. Subsequently, immunohistochemical testing revealed low expression of HER2. HER2 amplification and HER2 mutations have been reported in mucinous carcinomas of the ovary (17). However, to the best of our knowledge, HER2 expression has never been reported previously in ovarian LCNEC. The alteration of HER2 is an important oncogenic alteration that is relatively common in breast cancer and gastric cancers (18). A variety of drugs targeting HER2 molecular alterations are currently available, including small molecule TKIs, anti-HER2 antibodies, and antibody−drug conjugates (ADCs) (19, 20). T-DXd is a novel antibody−drug conjugate composed of an anti-HER2 antibody, a cleavable tetrapeptide-based linker, and a cytotoxic topoisomerase I inhibitor. T-DXd can accurately identify HER2-expressing tumor cells and release the cytotoxic drug exatecan derivative into the cells to kill the tumor more precisely and reduce systemic toxicity. It binds to the HER2 receptor on the surface of tumor cells through trastuzumab and enters the target cells through endocytosis. Subsequently, lysosomal enzymes cut the polypeptide linker and release the loaded DXd, which inhibits topoisomerase I activity and, in turn, initiates DNA damage and apoptosis to exert potent antitumor effects (21). Significantly, T-DXd also showed antitumor activity in patients with low HER2 (22). Fat-soluble DXd can penetrate cell membranes and enter nearby HER2-negative tumor cells, producing lethal effects. This is also called the bystander effect (23). Moreover, topoisomerase inhibitors such as etoposide and irinotecan are commonly used chemotherapeutic agents in LCNEC. Thus, T-DXd was selected for antitumor treatment. The tumor shrank significantly, and the physical performance of the patient was significantly improved after T-DXd treatment. Our case provides clinical evidence of the efficacy of T-DXd in ovarian LCNEC with low-level HER2 expression. In clinical work, doctors should give more precise treatment according to the patient’s disease condition, gene and other molecular changes.

## Conclusion

Ovarian neuroendocrine carcinoma is a rare but highly aggressive tumor. It is characterized by rapid progression, often accompanied by local infiltration and distant metastasis. We report a case of HER2-expressing ovarian LCNEC that responded well to T-DXd therapy. Our case supplements the limited literature, which helps clinicians better understand this rare disease and provides valuable treatment choices for advanced or recurrent ovarian LCNEC with low-level HER2 expression.

## Data availability statement

The original contributions presented in the study are included in the article/supplementary material. Further inquiries can be directed to the corresponding author.

## Ethics statement

Written informed consent was obtained from the individual(s) for the publication of any potentially identifiable images or data included in this article.

## Author contributions

YX: Writing – original draft, Writing – review & editing. YDC: Formal analysis, Resources, Writing – review & editing. XW: Writing – review & editing. YC: Formal analysis, Writing – review & editing. YW: Conceptualization, Writing – review & editing.
